# Measuring parental stress, illness perceptions, coping and quality of life in families of children newly diagnosed with autism spectrum disorder

**DOI:** 10.1192/bjo.2023.55

**Published:** 2023-05-18

**Authors:** Angelos Papadopoulos, Vassiliki Siafaka, Angeliki Tsapara, Dionysios Tafiadis, Konstantinos Kotsis, Petros Skapinakis, Meropi Tzoufi

**Affiliations:** Faculty of Medicine, School of Health Sciences, University of Ioannina, Ioannina, Greece; Department of Speech and Language Therapy, School of Health Sciences, University of Ioannina, Ioannina, Greece; Faculty of Medicine of Patras, School of Health Sciences, University of Patras, Patras, Greece

**Keywords:** Newly diagnosed children, autism spectrum disorder, family quality of life, parental stress, illness perceptions

## Abstract

**Background:**

A variety of psychosocial factors have been shown to affect the quality of life of families (FQoL).

**Aim:**

This study aimed to assess the impact of mother's demographic characteristics, parental stress, illness perceptions about autism spectrum disorder (ASD), coping strategies, ASD severity and time since diagnosis on FQoL during the initial period following diagnosis (≤6 months).

**Method:**

Fifty-three mothers of children newly diagnosed with ASD completed the Beach Center Family Quality of Life Scale, the Autism Parenting Stress Index, the Brief Illness Perception Questionnaire and the Brief Coping Orientation to Problems Experienced Inventory. A descriptive analysis was conducted on the demographic characteristics of the family. Eta coefficients and Pearson's analysis were used to determine the associations between the variables and the FQoL dimensions. Hierarchical regression was used to determine whether variables explained a statistically significant family quality of life variance.

**Results:**

Pearson's analysis and eta coefficients indicated several correlations. Hierarchical regression analysis showed that higher parental stress related to core autism symptoms was associated with poorer FQoL (95% CI −0.08 to −0.02, *P* = 0.001), and higher perceived treatment control was associated with better FQoL (95% CI 0.04–0.16, *P* = 0.001). In addition, stronger perceived personal control was associated with higher physical/material well-being (95% CI 0.01–0.16, *P* = 0.022) and higher disability-related support (95% CI 0.30–0.61, *P* = 0.001). Higher family monthly income was associated with better FQoL (95% CI 0.08–0.027, *P* = 0.000), whereas marital status (divorced mother) was correlated with poorer FQoL (95% CI −0.68 to −0.16, *P* = 0.002).

**Conclusions:**

Interventions should emphasise managing the disorder's characteristics and implementing psychoeducational and supportive programmes for parents, immediately after the diagnosis, to enhance FQoL.

The trend of increasing numbers of autism spectrum disorder (ASD) diagnoses in children highlights the need for early detection of this disorder and targeted interventions to improve the prognosis of the children.^1^ ASD is characterised by generalised limited–restricted interests and activities, a persistent impairment in social communication, and deviations in social–emotional functioning, and verbal and non-verbal communication skills.^2^ Having a family member with a disability is a huge daily challenge for the family.^3^ A variety of psychosocial factors have been shown to affect the quality of life of families (FQoL) with a child suffering from ASD, including (a) child-related factors: age, ASD severity, comorbid learning disability, emotional and behavioural difficulties; (b) parental factors: gender, parental stress, illness perceptions, coping styles, psychological distress and parental self-efficacy; and (c) other factors such as household income, employment status, social support, and support received by health and social services.^1,4^

In addition, various factors affect parental well-being, including impaired mental and physical health, social isolation and lack of family cohesion.^5^ Parents often experience significant practical problems with daily care activities and financial difficulties,^5,6^ resulting in poorer FQoL,^7^ and they report higher stress levels than parents of children with other disorders.^8^

According to Leventhal's common-sense model,^9^ when an individual experiences a health threat (e.g. symptoms or diagnosis), he or she forms cognitive and emotional representations of this threat. These representations play an important part in the choice of coping strategies that the individual adopts to manage stressful situations. Illness perceptions have been significantly investigated in physical health conditions but insufficiently in mental health conditions. To the best of the authors’ knowledge, studies about illness perceptions of parents of children with ASD have been particularly limited; however, these studies suggest that parents’ illness perceptions are associated with their depressive symptoms as well as with their decisions about treatment options.^10^ Moreover, evidence suggests that parents of preschoolers with developmental disorders report stronger beliefs about the disorder's chronic nature that could cause depressive symptomatology, especially during the first years after their child is diagnosed.^11^

As mentioned above, parents’ beliefs about the health problem of their children affect the coping mechanisms they adopt to deal with stressful situations. Parents characterise the diagnostic process as distressing, vague and difficult to understand, and they usually experience a variety of intense emotions, especially in the phase immediately after diagnosis.^12^ The literature regarding coping strategies adopted by parents of children recently diagnosed with ASD shows that most parents place themselves in the ‘resolved’ category regarding their child's diagnosis.^13^ Following the diagnosis, the parent's expectations for their child need to be altered and become more realistic based on the new situation.^13^ This modification necessitates a process that can cause intense emotional distress. Parents who accept their child's diagnosis manage to control their emotions and respond to their parental role and their child's needs, as ‘resolving’ the diagnosis allows the parental experience to be incorporated into an appropriate way of care.^13^

A qualitative study about the coping strategies of parents of children recently diagnosed with ASD indicated nine main coping mechanisms falling into three categories: adjusting to self-change, developing treatment strategies for the child with ASD and seeking support.^14^ The coping strategies adopted by parents of children with ASD (under the age of 5 years) differed significantly between the fathers and the mothers.^15^ Whereas mothers consistently reported using problem-focused and emotion-focused strategies, fathers generally reported more use of problem-focused strategies, indicating traditional gender roles as a factor that differentiates the adaptation process.^15^ In addition, according to the findings of a similar study, the main themes arising from the descriptions of mothers of their experience during the initial, challenging phase of diagnosis are guilt and blame, acceptance, focus on the present moment, future fear and confusion, competence, isolation and support.^16^

Taking all the above into consideration, the present study aimed to assess the impact of demographic characteristics of mothers, ASD severity, time since diagnosis, parental stress, illness perceptions about ASD and coping strategies on FQoL during the early period, within a maximum time of 6 months of the announcement of the diagnosis. With this study, we seek to contribute to a specific scientific field, covering a research gap in the Greek and international literature and concerning the consideration of all the above factors in a sample of mothers of children with a recent diagnosis of autism. We hypothesised that during the initial phase after diagnosis, the mothers of children with ASD would: (a) report a moderate level of parenting stress, (b) use adaptive coping mechanisms and (c) express emotional representations about the disorder. In addition, we hypothesised that FQoL would not be affected to a great extent in this initial phase and would be related to the severity of the child's condition, parental stress, mothers’ perceptions about ASD, their coping strategies, family income and family status.

## Method

### Participants

The sample consisted of 53 mothers of children with newly diagnosed ASD (with a maximum of 6 months from the diagnosis) who had visited a General Children's Hospital to receive the child's diagnosis by a child psychiatrist and were in follow-up every 2–3 months. The study sample was recruited from speech therapy centres and occupational therapy centres after an open call to the hospital, speech therapy centres and occupational therapy centres for parents receiving an autism diagnosis for their child.^17^ Parents who showed interest were contacted to participate in the study. Caregivers of newly diagnosed children with autism were chosen because they are a group with unique characteristics. They may not yet fully understand autism, the new demands of their parenting role and the need for enhanced childcare. Furthermore, they may not have been psychologically prepared to receive the diagnosis, or they may not have recovered from the shock of the diagnosis. The diagnosis was announced in person, and the doctor gave the parents all possible information, support and guidance. In the present study, only mothers participated, as they were the primary caregiver in all cases. The inclusion criteria were: recent (within 6 months) diagnosis of ASD in the child, absence of other family members with a disability, mother's ability to read and complete the questionnaires, and provision of direct care to the child. Of the 58 cases that met the inclusion criteria, two mothers declined participation in the study, and three cases were excluded from the analysis because of missing data. No participant withdrew from the study. All children whose mothers participated in this study were already involved in early speech therapy and occupational therapy intervention programmes. All participants were informed about the purposes and use of the results of this study, and written informed consent was received. The protection of the privacy of participants and the confidentiality of the data were ensured.

The authors assert that all procedures contributing to this work comply with the ethical standards of the relevant national and institutional committees on human experimentation and with the Helsinki Declaration of 1975, as revised in 2008. All procedures involving human subjects/patients were approved by the Scientific Committee of Karamandanio Children's Hospital, Patras, Achaia, Greece (approval number: 4173).

### Study instruments

The study design was cross-sectional, and all participants completed the following self-administered questionnaires.

The Beach Center FQoL scale was explicitly designed to assess the quality of life of families of children with disabilities.^18^ It includes 25 questions grouped into five subscales (family interaction, parenting, emotional well-being, physical/material well-being and disability-related support). The psychometric properties of the FQoL scale had excellent fit, *χ^2^* ^5^ = 3.4 1, *P* = 0.63. Comparative fit index = 1.00, root-mean-squared error associated 0.00, and Cronbach's α = 0.94. The five factors were found to be unidimensional and internally consistent with very good psychometric properties, and Cronbach's α ranged from α = 0.80 to α = 0.92 (family interaction: α = 0.92; parenting: α = 88; emotional well-being: α = 0.80; physical/material well-being: α = 0.88; and disability-related support: α = 0.92)^18^ The answers were given on a five-point Likert scale. The mean score for overall FQoL ranged from 1 to 5. The subscale scores and the mean total score were used in this study.^18,19^ The FQoL has been translated and validated in the Greek language.^20^

The Autism Parenting Stress Index (APSI) measures parental stress as perceived by parents or guardians of young children with ASD. It contains 13 common experiences that parents of children with ASD frequently encounter.^21^ Internal consistency estimates (Cronbach's α) were calculated (α = 0.82) for children with ASD. The index asks the parent to choose how stressful each item was for them using a five-point Likert scale from 0 (not stressful) to 4 (so stressful sometimes we feel we can't cope). The total score ranges from 0 to 52. APSI includes three domains: (a) core autism symptoms (social disability), (b) comorbid behaviours and (c) comorbid physical issues.^21^

The Brief Illness Perception Questionnaire (Brief-IPQ) assesses the parents’ cognitive and emotional representations of the disorder. The Brief-IPQ consists of nine dimensions: consequences, timeline, identity, personal control, treatment control, concerns, coherence, emotional representations and cause. Responses are given on a Likert scale from 0 to 10. Higher values are indicative of a stronger perception. The final item is an open-ended question requiring the participants to rank the three most important causal factors of the illness in their view. The Greek language version, with appropriate modification, was used in the study.^22,23^ All the subscales demonstrated very good internal consistency. The Cronbach α for each of the subscales ranged from α = 0.79 to α = 0.89.

The Brief-COPE inventory (Coping Orientation to Problems Experienced Inventory) assesses the coping strategies adopted by the parent. It contains 28 items, divided into 14 subscales: self-distraction, active coping, denial, substance use, emotional support, informational support, behavioural disengagement, venting, positive reframing, planning, humour, acceptance, religion and self-blame. The answers are given on a four-point Likert scale. Higher scores represent a more frequent use of each strategy. Cronbach's α values for the subscales of Brief-COPE ranged from α = 0.50 to α = 0.90.^24^ Brief-COPE has been translated and validated for the Greek population.^25^

### Statistical analysis

Descriptive analysis was conducted on the sociodemographic characteristics of the family and the demographic and clinical characteristics of the children. Mean values and standard deviations are reported for continuous variables and frequencies for categorical variables. For each questionnaire (FQoL, Brief-COPE and APSI), the mean scores of the dimensions and the total scores were computed according to the respective specifications and were treated as continuous variables. For the Brief-IPQ, each dimension was treated as a continuous variable. Eta was used to determine associations between categorical variables and the FQoL dimensions, as part of one-way analysis of variance. Associations between the dimensions of the Brief-IPQ, Brief-COPE, APSI and FQoL domains were examined using Pearson's correlation coefficient. A combined approach was used for hierarchical linear regression analysis (theoretical approach and strongly correlated items approach) to determine predictors for the final model. Hierarchical regression analysis was used to explain the most variability in the dependent variable (FQoL) with the fewest possible predictors. Hierarchical regression is an appropriate tool for analysis when variance of a criterion variable is explained by predictor variables that are correlated with each other. Hierarchical regression also allows the identification of the best predictor set of a pre-specified size and replicability, and the order of variable entry is determined by the researcher before the analysis is conducted. Thus, decisions are based on theory and research instead of being made arbitrarily, using blind automation, by the computer. The ‘Enter’ approach was used to select the final predictors. The variables of the hierarchical linear regression analysis models included monthly income, marital status, the age at which the child was able to combine two words, APSI dimensions and Brief-IPQ dimensions. In the model analysis for FQoL and subscales as dependent variables, seven covariates were examined, entered as follows: (a) predictors: (constant), monthly income, marital status; (b) predictors: (constant), monthly income, marital status, treatment control, personal control, consequences; (c) predictors: (constant), monthly income, marital status, treatment control, personal control, consequences, core autism symptoms; (d) predictors: (constant), monthly income, marital status, treatment control, personal control, consequences, core autism symptoms, denial. All statistical analyses were performed using SPSS version 25.

## Results

### Characteristics of the participants

The study sample consisted of 53 mothers with a mean age of 39.08 ± 4.43 years. The demographic characteristics of the study population are shown in Appendix A. The majority were married (79.2%), and 47.2% had completed university studies. The characteristics of the children are presented in Appendix B. Of the 53 children newly diagnosed with ASD, 42 were boys and 11 were girls, with a mean age of 4.49 ± 1.57 years (range 2.05–8.03 years). Regarding ASD severity, according to DSM-5, 26.4% (*N* = 14) of the children were categorised as level 1 (least severe), requiring support; 43.4% (*N* = 23) as level 2, requiring substantial support; and 30.2% (*N* = 16) as level 3, requiring very substantial support.

### Descriptive analysis of the questionnaires

The total mean score on the FQoL was 3.68 ± 0.59. The highest mean score was for the physical/material well-being domain (3.93 ± 0.79) (Table 1). The total mean score on APSI was 20.20 ± 8.65, corresponding to a medium stress level (Table 1). The mean scores on all the dimensions of the Brief-IPQ are presented in Appendix B. Perceptions of worry (illness concern) and the emotional effects of the disorder (emotional representations) were high (8.62 ± 1.86 and 8.60 ± 1.74, respectively). The scores on the Brief-COPE scale are shown in Appendix B. Coping strategies with the highest mean scores were planning (3.33 ± 0.67), acceptance (3.25 ± 0.69) and active coping (2.96 ± 0.83).

**Table 1:** Scores on the domains of the FQoL scale and APSI of mothers of children with ASD (*N* = 53)

	Minimum	Minimum	Mean	s.d.
FQoL				
Family interaction	1.83	5.00	3.85	0.75
Parenting	1.67	5.00	3.68	0.66
Emotional well-being	1.25	4.75	3.10	0.82
Physical/material well-being	1.60	5.00	3.93	0.79
Disability-related support	2.25	5.00	3.69	0.71
FQoL total score	1.96	4.96	3.68	0.59
APSI				
Core autism symptoms	1.00	18.00	10.92	4.11
Comorbid behaviours	0.00	15.00	4.98	3.80
Comorbid physical issues	0.00	16.00	4.30	3.36
APSI total score	2.00	40.00	20.20	8.65

FQoL, Beach Family Quality of Life; APSI, Autism Parenting Stress Index.

### FQoL associations

A descriptive analysis of FQoL scores by levels of each covariate of demographic and clinical characteristics is presented in Table 2. Specifically, the physical/material subscale of the FQoL was associated significantly with several covariates: (a) the age of mother, as the youngest mothers had higher mean FQoL scores: *F*(1, 51) = 4.59, *P* = 0.037; (b) the education of the mother, as the higher the level of education the better the FQoL: *F*(2, 50) = 3.88, *P* = 0.027; (c) employment status of the mother, as unemployed individuals and independent contractors had poorer FQoL: *F*(4, 48) = 2.81, *P* = 0.035; (d) relevant family medical history, as families with a history of mental illness reported significantly poorer FQoL compared with those without a history, and also with those with another type of medical history: *F*(3, 49) = 6.32, *P* = 0.001. In addition, marital status was associated significantly with FQoL total score, with divorced mothers reporting decreased FQoL compared with married mothers *F*(2, 50) = 13.62, *P* = 0.000. Family monthly income was also associated significantly with FQoL total score; the higher the income, the better the FQoL: F(4, 48) = 7.84, *P* = 0.000. The severity of the disorder did not seem to have any direct statistically significant correlation with FQoL.

**Table 2:** Univariate analysis of Beach FQoL scores by levels of each covariate of demographic and clinical characteristics

		Family	Parenting	Emotional	Physical/material	Disability-	FQoL
Covariate	Level	interaction		well-being	well-being	related	Total score
						support	
		*N*: mean (s.d.)	mean (s.d.)	*N*: mean (s.d.)	*N*: mean (s.d.)	*N*: mean (s.d.)	*N*: mean (s.d.)
Age of mother^a^	30–39 years	30: 3.86 (0.83)	30: 3.80 (0.65)	30: 3.27 (0753)	30: 4.13 (0.73)	30: 3.70 (0.70)	30: 3.75 (0.60)
	40–49 years	23: 3.84 (0.65)	23: 3.53 (0.65)	23: 2.88 (0.86)	23: 3.67 (0.80)	23: 3.68 (0.74)	23: 3.52 (0.57)
	*F*(1, 51)	0.004	2.20	3.13	4.59	0.006	1.951
	*P*-value	0.950	0.144	0.083	0.037	0.00	0.169
	η_p_^2^	0.00	0.41	0.58	0.08	0.940	0.37
Education of mother^a^	Gymnasium/preparatory	2: 4.41 (0.82)	2: 4.25 (0.35)	2: 3.25 (1.76)	2: 3.60 (1.41)	2: 3.50 (0.35)	2: 3.80 (0.94)
	High school	26: 3.71 (0.74)	26: 3.55 (0.72)	26: 2.83 (0.78)	26: 3.66 (0.92)	26: 3.62	26: 3.48
	University	25: 3.95 (0.76)	25 3.78 (0.58)	25: 3.37 (0.73)	25: 4.24 (0.45)	25: 3.78	25: 3.82
	*F*(2, 50)	1.21	1.49	2.92	3.88	0.366	2.26
	η_p_^2^	0.46	0.56	0.10	0.13	0.01	0.08
	*P*-value	0.30	0.23	0.06	0.02	0.69	0.11
Employment status of mother^a^	Unemployed	21: 3.71 (0.91)	21: 3.61 (0.62)	21: 3.03 (0.91)	21: 3.60 (0.82)	21: 3.60 (0.52)	21: 3.53 (0.58)
	Agriculturalist	3: 4.16 (0.00)	3: 3.83 (0.33)	3: 3.16 (0.76)	3: 4.20 (0.52)	3: 3.33 (0.52)	3: 3.74 (0.19)
	Independent contractor	2:3.66 (1.88)	2: 3.16 (2.12)	2: 2.50 (1.76)	2: 3.30 (2.40)	2: 4.00 (1.41)	2: 3.32 (1.91)
	Private employee	14: 4.02 (0.47)	14: 3.76 (0.61)	14: 3.08 (0.76)	14: 4.37 (0.34)	14: 3.60 (0.97)	14: 4.76 (0.53)
	Public employee	13: 3.85 (0.65)	13: 3.80 (0.60)	13: 3.30 (0.63)	13: 4.04 (0.63)	13: 3.75 (0.66)	13: 3.75 (0.53)
	F(4, 48)	0.501	0.524	0.492	2.816	0.355	0.550
	η_p_^2^	0.04	0.04	0.039	0.19	0.02	0.04
	*P*-value	0.73	0.71	0.74	0.035	0.83	0.70
Marital status^a^	Unmarried	2: 4.58 (0.35)	2: 4.25 (0.11)	2: 3.75 (0.70)	2: 4.10 (0.98)	2: 4.37 (0.88)	2: 4.21 (0.61)
	Married	42: 4.03 (0.58)	42: 3.78 (0.60)	42: 3.20 (0.78)	42: 4.10 (0.62)	42: 3.82 (0.64)	42: 3.79 (0.46)
	Divorced	9: 2.85 (0.67)	9: 3.12 (0.69)	9: 2.50 (0.78)	9: 3.08 (0.97)	9: 2.91 (0.45)	9: 2.89 (0.59)
	F(2, 50)	16.04	5.01	3.70	7.82	9.13	13.62
	η_p_^2^	0.30	0.16	0.12	0.23	0.26	0.35
	*P*-value	0.000	0.010	0.032	0.001	0.000	0.000
Family monthly income^a^	0–400	6: 2.94 (0.95)	6: 3.08 (0.86)	6: 2.08 (0.54)	6: 2.26 (0.51)	6: 3.08 (0.30)	6: 2.69 (0.20)
	401–800	15: 3.68 (0.82)	15: 3.58 (0.65)	15: 3.03 (0.74)	15: 3.76 (0.47)	15: 3.80 (0.48)	15: 3.57 (0.10)
	801–1500	16: 4.08 (0.67)	16: 3.91 (0.56)	16: 3.14 (0.75)	16: 4.31 (0.53)	16: 3.75 (0.92)	16: 3.84 (0.15)
	1501–2500	10: 4.18 (0.19)	10: 3.96 (0.50)	10: 3.57 (0.68)	10: 4.28 (0.26)	10: 3.87 (0.73)	10: 3.97 (0.11)
	>2500	6: 4.02 (0.35)	6: 3.47 (0.570	6: 3.41 (0.87)	6: 4.46 (0.41)	6: 3.58 (0.71)	6: 3.79 (0.15)
	F(4, 48)	4.09	2.73	4.24	26.02	1.44	7.84
	η_p_^2^	0.25	0.18	0.26	0.68	0.10	0.39
	*P*-value	0.06	0.39	0.05	0.000	0.23	0.000
Relevant	Developmental disorder	12: 3.97 (0.38)	12: 3.66 (0.73)	12: 3.27 (0.71)	12: 4.15 (0.49)	12: 3.79 (0.65)	12: 3.77 (0.39)
Family	Mental disorder	10: 3.51 (0.88)	10: 3.50 (0.82)	10: (2.90 (0.92)	10: 3.08 (1.07)	10: 3.32 (0.54)	10: 3.26 (0.75)
Medical	Neurological disorders	1: 4.16 (-)	1: 3.83 (-)	1:3.50 (-)	1: 4.40 (-)	1: 3.32 (-)	1:3.98 (-)
History^a^	None	30: 3.91 (0.81)	30: 3.74 (0.59)	30: 3.09 (0.84)	30: 4.12 (0.60)	30: 3.76 (0.77)	30:3.72 (0.58)
	F(3, 49)	0.87	0.37	0.43	6.32	1.13	1.91
	η_p_^2^	0.05	0.02	0.02	0.27	0.06	0.10
	*P*-value	0.45	0.76	0.72	0.01	0.34	0.13
Gender of child^a^	Boy	42: 3.81 (0.80)	42: 3.62 (0.70)	42: 3.14 (0.86)	42: 3.90 (0.84)	42: 3.72 (0.68)	42: 3.64 (0.63)
	Girl	11: 4.01 (0.52)	11: 3.93 (0.41)	11: 2.95 (0.62)	11: 4.03 (0.58)	11: 3.56 (0.85)	11: 3.70 (0.46)
	F(1, 51)	0.62	2.03	0.45	0.22	0.42	0.085
	η_p_^2^	0.01	0.38	0.009	0.004	0.008	0.39
	*P*-value	0.43	0.16	0.50	0.64	0.51	0.77
Attention deficit^a^	Yes	46: 3.80 (0.79)	46: 3.67 (0.68)	46: 3.05 (0.85)	46: 3.90 (0.83)	46: 3.71 (0.72)	46: 3.63 (0.63)
	No	07: 4.19 (0.17)	07: 3.80 (0.45)	07: 3.39 (0.53)	07: 4.143 (0.44)	07: 3.53 (0.63)	07: 3.81 (0.29)
	F(1, 51)	1.62	0.26	1.00	0.54	0.38	0.56
	η_p_^2^	0.03	0.05	0.01	0.01	0.008	0.01
	*P*-value	0.20	0.60	0.32	0.46	0.53	0.45
ASD severity^a^	Level 1	14: 4.22 (0.62)	14: 3.97 (0.60)	14: 3.23 (1.04)	14: 4.07 (0.96)	14: 4.00 (0.51)	14: 3.90 (0.63)
	Level 2	23: 3.84 (0.59)	23: 3.65 (0.68)	23: 3.06 (0.75)	23: 3.94 (0.72)	23: 3.64 (0.70)	23: 3.63 (0.54)
	Level 3	16: 3.54 (0.92)	16: 3.47 (0.62)	16: 3.04 (0.73)	16: 3.80 (0.74)	16: 3.50 (0.82)	16: 3.47 (0.60)
	F(2, 50)	3.38	2.24	0.22	0.43	2.00	2.01
	η_p_^2^	0.13	0.02	0.02	0.04	0.03	0.02
	*P*-value	0.052	0.11	0.79	0.65	0.14	0.14

a. Eta coefficient test

ASD, autism spectrum disorder; FQoL, BEACH Family Quality of Life.

Table 3 presents in detail the associations between the mean scores on the dimensions of the Brief-IPQ, Brief-COPE and APSI and the mean scores on the subscales and total score of FQoL. Specifically, concerning the Brief-IPQ dimensions, the total FQoL score was negatively associated with beliefs about the consequences of the disorder (*P* < 0.001), illness concerns (*P* < 0.010) and emotional representations (*P* < 0.050), whereas it was positively associated with perceived treatment control (*P* < 0.001) and beliefs about the coherent understanding of the disorder (*P* < 0.050). Regarding coping strategies (Brief-COPE), the total FQoL score was negatively associated with denial (*P* < 0.050) and positively associated with emotional support (*P* < 0.050), positive reframing (*P* < 0.050) and humour (*P* < 0.050). Moreover, higher autism parenting stress levels (APSI) were correlated with poorer FQoL (*P* < 0.001).

**Table 3:** ΤZero-order correlations between scores on Brief-IPQ, Brief-COPE, APSI and FQoL of mothers of children with autism spectrum disorder

	BEACH FQoL					
	Family interaction	Parenting	Emotional well-being	Physical/material well-being	Disability-related support	Total FQoL
Brief-IPQ^1^						
Consequences	−0.331^2^	NS	−0.434**	−0.347^2^	−0.474***	−0.467***
Timeline	NS	NS	NS	NS	−0.330^2^	NS
Personal control	NS	NS	NS	NS	0.328^2^	NS
Treatment control	0.419**	0.643***	0.450**	NS	0.484***	0.556***
Identity	NS	NS	NS	NS	NS	NS
Illness concern	−0.344^2^	−0.344^2^	−0.386**	−0.293^2^	−0.410**	−0.442**
Coherence			0.360**	NS	0.328^2^	0.345^2^
Emotional representations	−0.374**	−0.286^2^	NS	NS	−0.288^2^	−0.328^2^
Causes	NS	NS	NS	NS	NS	NS
Brief-COPE^1^						
Self-distraction	NS	NS	NS	NS	NS	NS
Active coping	NS	NS	0.305^2^	NS	NS	NS
Denial	NS	−0.330^2^	−0.298^2^	NS	NS	−0.308^2^
Substance use	NS	NS	0.281^2^	NS	NS	NS
Emotional support	NS	NS	0.273^2^	0.366**	NS	0.282^2^
Informational support	NS	NS	0.330^2^	NS	NS	NS
Behavioural disengagement	NS	NS	NS	NS	−0.311^2^	NS
Venting	NS	NS	0.316^2^	NS	0.311^2^	NS
Positive reframing	NS	0.324^2^	0.305^2^	NS	NS	0.301^2^
Planning	NS	NS	NS	NS	NS	NS
Humour	NS	0.320^2^	NS	NS	0.367**	0.326^2^
Acceptance	NS	NS	NS	NS	NS	NS
Religion	NS	NS	NS	NS	0.361**	NS
Self-blame	NS	NS	NS	NS	NS	NS
APSI^1^						
Core autism symptoms	−0.487***	−0.484***	−0.549***	NS	−0.555***	−0.571***
Comorbid behaviours	NS	NS	NS	NS	−0.296^2^	−0.299^2^
Comorbid physical issues	NS	−0.457**	NS	−0.519***	NS	−0.377**
APSI total score	−0.449**	−0.461**	−0.435**	−0.414**	−0.440**	−0.549***

The Eta coefficient test was applied to all demographics in the table. FQoL, BEACH Family Quality of Life; Brief-IPQ: Brief Illness Perception Questionnaire; Brief-COPE, Brief COPE Inventory; APSI, Autism Parenting Stress Index; NS, not significant.

1 Pearson correlation coefficients.

2 *P* < 0.05, **P < 0.01, ***P < 0.001.

### Hierarchical regression analysis

In the hierarchical regression analysis (Table 4), it was evident that core autism symptoms (APSI) had negative associations with total FQoL score (β = −0.053, *P* = 0.001) and its subscales: family interaction (β = −0.078, *P* = 0.002), parenting (β = −0.048, *P* = 0.032), emotional well-being (β = −0.071, *P* = 0.014) and disability-related support (β = −0.073, *P* = 0.002). Regarding the associations between Brief-IPQ dimensions and FQoL, a high score for perceived treatment control was associated with a high total FQoL score (β = 0.104, *P* = 0.001) and high scores on the parenting (β = 0.188, *P* = 0.000) and emotional well-being (β = 0.127, *P* = 0.027) subscales. In addition, perceived personal control was positively associated with physical/material well-being (β = 0.089, *P* = 0.022) and disability-related support (β = 0.067, *P* = 0.045).

**Table 4:** Statistically significant effects of parenting stress (APSI), Illness Perceptions (Brief-IPQ), and coping strategies (Brief-COPE) of the mothers of children with autism spectrum disorder (ASD) on family quality of life (FQoL) (*N* = 53)

		FQoL				
		Family interaction				
		β	*t*	Sig.	s.e.	95% CI
R^2^ = 0.542	Marital status	−0.837	−4.310	0.000	0.194	−1.22, −0.44
	Monthly family income	0.161	2.272	0.028	0.071	0.01, 0.30
	Core autism symptoms (APSI)	−0.078	−3.377	0.002	0.023	−0.12, −0.03
		Parenting				
R^2^ = 0.480	Treatment control (Brief-IPQ)	0.188	4.372	0.000	0.043	0.10, 0.27
	Core autism symptoms (APSI)	−0.048	−2.208	0.032	0.022	−0.09, −0.00
		Emotional well-being				
R^2^ = 0.432	Monthly family income	0.225	2.628	0.012	0.085	0.05, 0.39
	Treatment control (Brief-IPQ)	0.127	2.280	0.027	0.056	0.01, 0.24
	Core autism symptoms (APSI)	−0.071	−2.551	0.014	0.028	−0.12, −0.015
		Physical/material well-being				
R^2^ = 0.514	Monthly family income	0.457	5.992	0.000	0.076	0.30, 0.61
	Personal control (Brief-IPQ)	0.089	2.369	0.022	0.037	0.01, 0.16
		Disability-related support				
R^2^ = 0.546	Personal control (Brief-IPQ)	0.067	2.066	0.045	0.183	0.00, 0.13
	Core autism symptoms (APSI)	−0.073	−3.344	0.002	0.033	−0.11, −0.02
	Marital status	−0.580	−3.176	0.003	0.022	−0.94, −0.21
		FQoL total score				
R^2^ = 0.681	Marital status	−0.424	−3.309	0.002	0.128	−0.68, −0.16
	Monthly family income	0.176	3.779	0.000	0.047	0.08, 0.27
	Treatment control (Brief-IPQ)	0.104	3.427	0.001	0.030	0.04, 0.16
	Core autism symptoms (APSI)	−0.053	−3.498	0.001	0.015	−0.08, −0.02

R^2^ adj, R^2^ adjusted; β, unstandardised coefficient B. *P*-values for quality-of-life outcomes after hierarchical regression analysis. FQoL, Beach Center Family Quality of Life Scale; Brief-IPQ, Brief Illness Perceptions Questionnaire; APSI, Autism Parenting Stress Index.

Finally, marital status and family monthly income significantly affected FQoL. Specifically, higher family monthly income was associated with higher total FQoL score (β = 0.176, *P* = 0.000) and higher family interaction (β = 0.161, *P* = 0.028), emotional well-being (β = 0.225, *P* = 0.012), and physical/material well-being (β = 0.457, *P* = 0.000). In addition, marital status had statistically significant associations with total FQoL score (β = −0.424, *P* = 0.002) and family interaction subscale (β = −0.837, *P* = 0.000), indicating that single-parent families (divorced mothers) had lower FQoL scores. More details are provided in Table 4.

## Discussion

This study aimed to assess levels of parental stress, mothers’ perceptions about ASD, the coping mechanisms they adopted in the initial period after diagnosis, and the impact of these factors on FQoL. Mothers of children with newly diagnosed ASD (within 6 months) reported a moderate level of FQoL, as revealed by the assessment in the initial post-diagnosis period. In addition, FQoL was associated with parental stress, beliefs about the controllability of ASD, parents’ marital status and family income.

According to the findings, mothers experienced moderate stress levels in the first few months (<6 months) following the reception of an ASD diagnosis, consistent with findings from other studies.^21,26^ Usually, parents are already very concerned about their children's development before they are notified of the diagnosis of ASD, because they have noticed deficiencies in social transactions and communication.^27^

Regarding FQoL, the total mean score was at a moderate level, consistent with the literature.^5,28,29^ In this study, the primary caregiver was the mother, who focused heavily on the child with the disability, which often results in the other family members being sidelined.^30^ Mothers appear to be less satisfied with their everyday life. Parents, especially mothers, are unhappy about the lack of time for their interests because they do not have appropriate external help to take care of the individual needs of family members and relieve their distress.^31^

With regard to the associations between psychological factors and FQoL, hierarchical regression analysis demonstrated significant negative associations between the core autism symptoms domain (APSI) and the total mean FQoL score and scores on the family interaction, parenting, emotional well-being and disability-related support domain; the higher the stress experienced by the mother, the poorer the FQoL, a finding that is in line with those of other studies.^5^ A large body of evidence indicates that FQoL is closely related to specific symptoms, especially communication disorders, difficulties in social interaction and behavioural problems.^32^ To the best of the authors’ knowledge, the impact of parental stress as perceived by parents of young children newly diagnosed with ASD on FQoL has not been previously investigated. Our findings, however, are consistent with other reports on the effects of parental stress, regardless of the time elapsed since the diagnosis.^33^ Parental stress has been identified as a mediator between the child's problems and parenting strategies.^34^ In addition, the parent's mental health can affect the quality of interactions with their child^34^ and the FQoL.^35^ Specifically, a mother's level of parenting stress and depressive symptoms influences family interactions, in particular, mother–child and father–child relationships.^36^

In the present study, investigation of mothers’ perceptions about ASD revealed serious concerns and negative thoughts about their children's health problem and its consequences, with intense emotional impact (e.g. experiencing fear and sadness), findings that are in line with those of previous studies.^10,28^ Mothers can identify many symptoms and attribute them to their children's ASD.^37^ Moreover, most mothers sufficiently understand the nature of their children's health problems, even when the period from diagnosis was is than 6 months. Parents may have received adequate information immediately after diagnosis about the characteristics of the disorder and how to manage their child's symptoms.^10^

Hierarchical regression analysis demonstrated that the mothers’ perceptions about treatment effectiveness affected total FQoL score and two domains. All the children in the study sample had been enrolled in intervention programmes, such as speech therapy and occupational therapy, which probably raised mothers’ expectations of improvement in their children's symptoms. The timing of the diagnosis and the provision of support immediately after diagnosis (e.g. the start of an intervention programme) have been shown to positively affect parental satisfaction.^38^

Furthermore, perceived personal control was positively associated with the physical/material well-being and disability-related support dimensions of the FQoL. A similar study of parents’ perceptions about ASD and their influence on treatment choice suggested that higher personal control was correlated with a lower likelihood of choice of biomedical and psychotropic treatment and a more comprehensive search for alternative forms of intervention and support.^39^

Regarding coping strategies, mothers during the initial period (first 6 months) after diagnosis often used adaptive coping strategies, such as planning and acceptance, to deal with the situation; these are coping mechanisms commonly used by parents according to similar studies.^40^ In the time immediately after diagnosis, it seems that parents endeavour to understand the disorder, seeking informational support^41^ and trying to find meaning in the stressful situation, with positive reframing and emotional support from others.^13^ Research data indicates that parents’ stress levels drop after receiving an accurate diagnosis.^42^ They seem to be better able to accept autism and the limitations it imposes on their family life, and adjust and have more reasonable hopes for their child's future as a result of this. Moreover, a brief cognitive behavioural intervention, problem-solving education, has been suggested to be valuable during the period immediately following a child's diagnosis of ASD (3 months after the diagnosis).^43^

Problem-focused coping strategies and seeking emotional support appear to reduce parental stress and improve FQoL.^44^ Receiving social support allows parents to be more flexible in working and meeting the family's financial needs (physical/material well-being) and reduces the risk of mental health problems.^45^ Similarly, when there is support from communities (e.g. friends, community services, professionals), caregivers experience overall greater life satisfaction.^46^

In addition, based on the results of multivariable regression analyses, associations were observed between both monthly family income and marital status and total FQoL score and some FQoL domains. Few studies have focused on these two factors concerning FQoL in the immediate post-diagnosis period. Financial resources can provide greater comfort, and parents usually require financial support to ensure access to leisure, health and transportation, which have essential roles in FQoL.^47^ Raising a child with ASD has a significant impact on family finances and negatively affects family life.^48^ As a result, families with a higher income have more options for coping with the additional health and everyday demands of members with disabilities.^20^ In addition, single-parent status, as expected, had a direct negative impact on FQoL, as raising a child as a single parent is a difficult situation that is compounded by the diagnosis of ASD^49^

Our hypothesis that mothers of children with ASD would report a moderate amount of parenting stress in the initial time after diagnosis was confirmed by this study. They used mainly positive coping mechanisms, such as acceptance and planning, and expressed emotional representations of the disorder as expected. Regarding the hypothesis concerning the FQoL immediately after the diagnosis, the study results confirmed our expectations: the FQoL was at a moderate level in the early time after diagnosis and was correlated with parental stress, mothers’ beliefs about the controllability of ASD, family status and family income. However, the severity of the disorder did not seem to have any direct effect on FQoL. It is more likely that this relationship is mediated by mothers’ parental stress, as ASD severity is associated with parental stress levels, which affect FQoL. Additional analysis showed a moderate negative correlation between APSI and FQoL mediated by severity. There have been conflicting findings of studies on the effect of ASD severity on FQoL; some studies concluded that FQoL is negatively affected by the characteristics of the disorder,^2^ whereas other studies indicated that the severity of ASD did not significantly affect FQoL,^50^ suggesting that the symptom severity is not the primary factor affecting parents’ quality of life or other indicators of well-being.

### Clinical implications

Specific clinical implications emerge from the findings of the present study. Assessment of the emotional burden of parents of children with ASD and identification and understanding of their perceptions about ASD and the defence mechanisms they adopt, especially in the initial period after diagnosis, are crucial to the implementation of appropriate intervention programmes. In order to significantly improve the quality of life of children with ASD and their families, parents need prompt, detailed and substantial information about the nature of the disorder, its symptoms and the available treatment interventions to be able to manage their daily challenges effectively and to participate in the decision-making process. On identification of pronounced parental stress, dysfunctional perceptions and maladaptive defence mechanisms, parents should be referred for assessment and intervention by mental health professionals to help reduce their symptoms of stress, anxiety and depression and improve their self-efficacy and parental care skills. Better parental understanding of the child's disorder and management of the emotions it causes can positively improve FQoL.

### Limitations and strengths

This study had certain limitations that should be kept in mind. First, given the inclusion criteria and the incidence of ASD, it was only possible to recruit a relatively small number of mothers; this limits the generalisability of the results, and the findings of the study should be further confirmed using large-sample analysis. Second, as the present study was cross-sectional, it is impossible to comment on cause–effect relationships between the assessed factors. Prospective assessment should be used to investigate possible changes in parental psychological distress, illness perceptions and coping strategies and their impact on FQoL. Moreover, the duration of treatment and how it affects the stress level of the family could be studied in future research, using a control group that has not yet received treatment. However, the study's main strength was the assessment of beliefs, emotions and ways of coping adopted by mothers in the initial period after diagnosis of ASD in their children. More specifically, to the best of our knowledge, the stress experienced by mothers with a child newly diagnosed with ASD and the coping strategies they adopt, as well as the impact of these factors on FQoL, has not previously been adequately studied.

### Findings and future work

In summary, the mothers of children with newly diagnosed ASD reported a moderate FQoL, according to the assessment in the initial post-diagnosis period. In addition, FQoL was associated with parental stress, beliefs about the controllability of the ASD, marital status and family income. The findings of this study confirm that in planning interventions, emphasis should be placed not only on the characteristics of ASD but also on parents’ psychological factors that affect FQoL. Holistic, multidisciplinary intervention should include psychoeducational and supportive programmes for parents, designed to improve the emotional well-being of children with ASD and their parents and to reinforce FQoL. Finally, it will be of importance to confirm the findings of the study based on a large-sample analysis.

## Data Availability

The data that support the findings of this study are available on request from the corresponding author, A.P. The data are not publicly available owing to restrictions as they contain information that could compromise the privacy of research participants.

## Appendix A Demographic characteristics of the families of children with autism spectrum disorder (*N* = 53)

**Table:**

**Mother's age in years,** mean ± s.d. (range)	39.08 ± 4.43 (31–49)
**Mother's age group**	
30–39, *N* (%)	30 (56.6)
40–49, *N* (%)	23 (43.4)
**Marital status**, *N* (%)	
Married	42 (79.2)
Divorced	9 (17)
Unmarried	2 (3.8)
**Mother's education level**, *N* (%)	
Gymnasium school (preparatory high school)	2 (3.8)
High school	26 (49.1)
University degree	25 (47.2
**Mother's profession**, *N* (%)	
Economically inactive	21 (39.6)
Farmer	3 (5.7)
Private employee	14 (26.4)
Civil servant	13 (24.5)
Freelancer	2 (3.8)
**Relevant family medical history**, *N* (%)	
Developmental disorder	12 (22.6)
Mental disorders	10 (18.9)
Neurological disorder	1 (1.9)
None	30 (56.6)
**Monthly family income (euro)**	
0–400	6 (11.3)
401–800	15 (28.3)
801–1.500	16 (30.2)
1.501–2.500	10 (18.9)
2.500+	6 (11.3)

## Appendix B Scores on the Brief-IPQ and Brief-COPE questionnaires of mothers of children with autism spectrum disorder (*N* = 53)

**Table:**

Brief-IPQ				
	Minimum	Maximum	Mean	s.d.
Consequences	1.00	10.00	7.39	2.31
Timeline	1.00	10.00	6.75	2.95
Personal control	1.00	10.00	6.56	2.24
Treatment control	2.00	10.00	7.96	1.74
Identity	.00	9.00	6.90	2.17
Illness concern	4.00	10.00	8.62	1.86
Coherence	2.00	10.00	7.83	2.09
Emotional representations	4.00	10.00	8.60	1.74
Cause^1^, frequency (%)				
Emotional cause	5 (9.4%)			
Behavioural cause	10 (18.9%)			
Risk factors	13 (24.5%)			
External factors	12 (22.6%)			
Brief-COPE				
	Minimum	Maximum	Mean	s.d.
Self-distraction	2.00	8.00	5.01	1.42
Active coping	2.00	8.00	5.92	1.66
Denial	2.00	8.00	4.32	1.59
Substance use	2.00	8.00	2.03	0.27
Emotional support	2.00	8.00	5.47	1.51
Informational support	2.00	8.00	5.71	1.40
Behavioural disengagement	2.00	8.00	3.07	1.45
Venting	2.00	8.00	5.43	1.47
Positive reframing	2.00	8.00	5.71	1.79
Planning	2.00	8.00	6.67	1.35
Humour	2.00	6.00	2.98	1.16
Acceptance	4.00	8.00	6.50	1.38
Religion	2.00	8.00	4.37	1.96
Self-blame	2.00	8.00	5.54	1.90

Brief-IPQ: Brief-Illness Perception Questionnaire; Brief-COPE: Brief Coping Orientation to Problems Experienced Inventory.

1 Perceived cause as reported by mothers.
